# A Single-Wavelength Near-Infrared Photoacoustic Spectroscopy for Noninvasive Glucose Detection Using Machine Learning

**DOI:** 10.3390/bioengineering13040444

**Published:** 2026-04-10

**Authors:** Abdulrahman Aloraynan, Eunice Chu, Jishen Wang, Dawood Alsaedi, Dayan Ban

**Affiliations:** 1 Department of Electrical Engineering, Umm Al-Qura University, Makkah 21955, Saudi Arabia; 2 Department of Electrical and Computer Engineering, University of Waterloo, 200 University Ave W, Waterloo, ON N2L 3G1, Canada; eunice.chu@uwaterloo.ca (E.C.);; 3 Waterloo Institute for Nanotechnology, University of Waterloo, 200 University Ave W, Waterloo, ON N2L 3G1, Canada; 4 Electrical Engineering Department, Taif University, Taif 26571, Saudi Arabia

**Keywords:** noninvasive glucose detection, photoacoustic spectroscopy, near-infrared spectroscopy, machine learning

## Abstract

According to the International Diabetes Federation, 589 million adults worldwide live with diabetes in 2025 (approximately 1 in 9 adults). The development of convenient noninvasive blood glucose monitoring systems has been a central focus in diabetes management. Optical spectroscopy has advanced significantly among all noninvasive glucose detection techniques. A photoacoustic system has been developed using a single-wavelength near-infrared laser, operating at 1625 nm, where glucose exhibits an overtone absorption band with relatively low water interference. The noninvasive system has been evaluated using artificial skin phantoms, with different glucose concentrations, covering both normoglycemic and hyperglycemic blood glucose levels. The detection sensitivity of the developed system has been enhanced to ±15 mg/dL across the entire clinically relevant glucose range. K-nearest neighbours and wide neural network machine learning models were developed for noninvasive glucose classification. The models achieved prediction accuracies of 80.0% and 81.5%, respectively, with 100% of the predicted data located within zones A and B of Clarke’s error grid analysis. These findings satisfy the regulatory requirements for glucose monitors established by Health Canada and the U.S. Food and Drug Administration.

## 1. Introduction

Researchers have explored various techniques for noninvasive glucose monitoring, including optical spectroscopy [1,2,3,4], thermal emission spectroscopy [5,6], electromagnetic wave sensing [7,8], bioimpedance spectroscopy [9,10], and photoacoustic (PA) spectroscopy [11,12]. Optical spectroscopy has been highly advanced due to its direct correlation with the absorption bands of the molecular structure of glucose (C_6_H_12_O_6_) [13,14]. Infrared (IR) spectroscopy, including both mid-infrared (MIR) [15,16] and near-infrared (NIR) regions [17,18], exhibits distinct and broad glucose absorption features, highlighting its potential to be used in noninvasive glucose sensing applications. Blood glucose levels can be noninvasively measured using optical methods through human blood vessels [19,20,21] or via the interstitial fluid (ISF) in human skin [22,23,24]. Nevertheless, using optical spectroscopy as a standalone technique encounters significant limitations, including light scattering, reflection, refraction, and spectral interference from other blood constituents.

The integration of IR and PA spectroscopy has emerged as a promising approach to overcome the limitations of optical techniques [25,26,27]. Both MIR and NIR lasers can be used as optical sources to generate acoustic signals. Quantum cascade lasers (QCLs) operating in the MIR region can generate strong acoustic signals due to their high pulsed power, where glucose exhibits distinct molecular fingerprints. However, MIR radiation has a superficial penetration depth in human skin due to its high water absorption. This restricts the ability of MIR light to reach blood vessels and acquire real-time glucose information [28]. In contrast, NIR spectroscopy is a relatively cost-effective approach that has the advantage of compact system configurations. Furthermore, short wavelengths within the NIR region (780–2500 nm) can penetrate deeper (>1 mm) into skin and can reach superficial vasculature due to weaker water absorption [29]. Within this range, the first overtone absorption band of glucose near 1638 nm can be exploited for glucose detection. Table 1 shows the absorption peaks of the glucose functional groups in the NIR region [7].

The integration of NIR and PA spectroscopy for glucose detection was first reported in 1993 by Christison et al. [30], where a wavelength of 967 nm was employed. In this study, a linear correlation of the PA signals to the physiological glucose concentrations in whole blood samples was found. Subsequently, the same research group suggested using NIR wavelengths from 1550 to 1750 nm to enhance the sensitivity of the photoacoustic glucose response in gelatin-based tissue phantoms [31].

Zhang et al. [32] developed an NIR and PA technique utilizing both peak-to-peak amplitude and time delay information at a wavelength of 1650 nm, achieving an average root mean square error (RMSE) of 31.5 mg/dL in glucose solutions. Ghazaryan et al. [33] employed an extended NIR range from 850 to 1900 nm to investigate the ratiometric approach between two wavelengths in order to enhance the signal-to-noise ratio (SNR). A positive correlation with glucose was found in the range of 1536–1688 nm using dictionary learning methods. Similarly, Tanaka et al. [34] adopted differential continuous wave PA spectroscopy (DCW-PAS) using wavelengths of 1382 and 1610 nm to mitigate water absorption effects. In this study, a correlation coefficient of 0.8 was achieved with a standard error of 48 mg/dL for in vivo earlobe measurements. Friedlein et al. [35] introduced a dual-comb PA spectroscopy technique, which allows simultaneous photoacoustic measurement using multi-wavelength and enhances the SNR.

**Figure 1:**
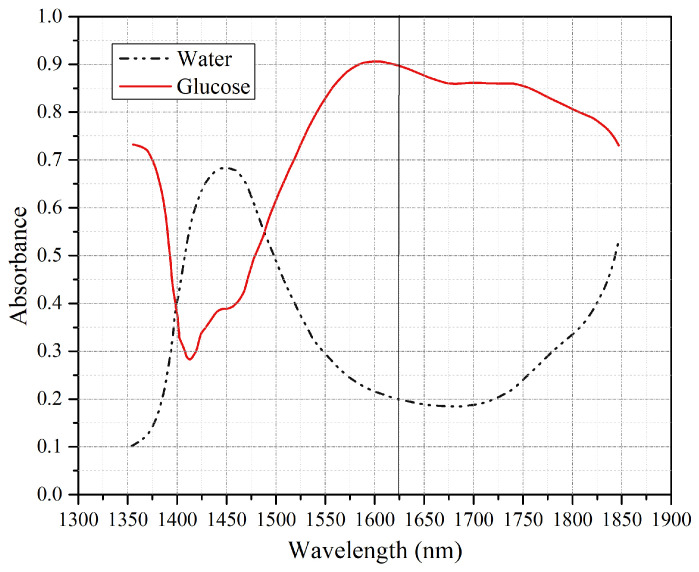
Glucose absorption compared to water absorption in the NIR region [40].

Tissue-mimicking phantoms with varying glucose concentrations were studied with an ultrasound-modulated optical sensing system using a wavelength of 1645 nm by Park et al. [36]. A mean absolute relative difference (MARD) of 26.6% was obtained for glucose levels ranging from 0 to 400 mg/dL. Yang et al. [37] presented a low-power NIR continuous-wave laser operating from 1500 to 1630 nm for glucose solution measurements, yielding an RMSE of 13.97 mg/dL after applying different regression models. RMSE was then reduced to 10.94 mg/dL by Prasad V et al. [38] at a wavelength of 905 nm. Recently, Zhang et al. [39] reported an RMSE of 6.07 mg/dL using a pulsed laser diode operating at 1064 nm. In general, the NIR wavelength range between 1550 and 1750 nm has been widely adopted due to its relatively lower water absorption compared to glucose, as illustrated in Figure 1. In particular, the glucose overtone absorption band between 1600 and 1650 nm is well suited for noninvasive detection systems.

Despite extensive research on NIR–PA spectroscopy for noninvasive glucose monitoring, no clinically approved devices have yet been realized. This limitation is primarily attributed to inadequate detection sensitivity and selectivity for clinically approved glucose monitors. Regulatory agencies, including the U.S. Food and Drug Administration (FDA) and Health Canada, require a detection accuracy of ±15 mg/dL, with at least 99% of the results falling within zones A and B of the Clarke error grid analysis (EGA) [41,42]. One of the key open challenges in NIR–PA glucose detecting is achieving accurate and stable measurements, without the need for advanced algorithms or extensive post-processing for the data. Although multi-wavelength and hyperspectral methods provide more spectral information, they increase system complexity, cost, and processing requirements. In contrast, a single-wavelength NIR-PA approach offers a simpler sensing configuration. Therefore, an investigation of the single-wavelength ability to achieve clinically relevant glucose discrimination with minimum data processing is important to balance sensing performance with practical system simplicity in noninvasive glucose monitoring.

In this paper, a single-wavelength NIR–PA system is developed for noninvasive blood glucose monitoring. A short-wavelength-infrared (SWIR) diode laser operating at a glucose overtone of 1625 nm is employed to detect glucose levels in biomedical skin phantoms with optical properties similar to human skin. The glucose concentrations in these phantoms span the clinically relevant range for both normoglycemic individuals and those living with diabetes. The detection limit of the PA and NIR system has improved to ±15 mg/dL by introducing a dominant spectral peak-based feature extraction approach. K-nearest neighbours (KNN) and wide neural network (WNN) machine learning models were developed for noninvasive glucose classification. These models achieved prediction accuracies of 80.0% and 81.5%, respectively, with 100% of the predicted results falling within zones A and B of Clarke’s EGA. These findings fulfill the regulations for glucose monitors established by Health Canada and the U.S. Food and Drug Administration.

## 2. Materials and Methods

**Figure 2:**
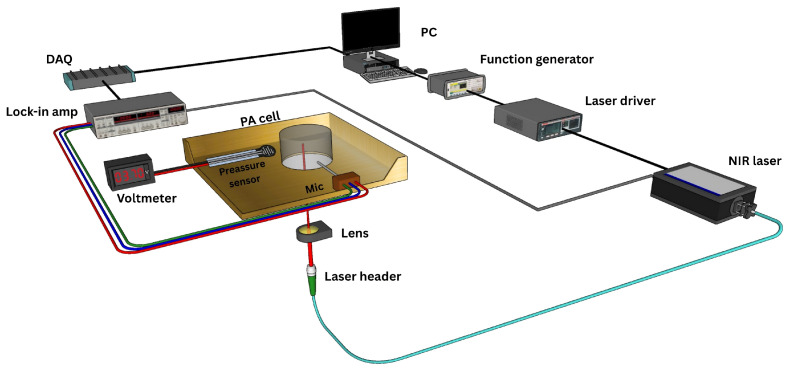
Schematic of the setup used for glucose detection using NIR and PA spectroscopy.

The schematic of the single-wavelength NIR-PA system developed for noninvasive glucose detection is shown in Figure 2. A short-wavelength infrared (SWIR) diode laser (FPL1054P, Thorlabs, Newton, NJ, USA) was employed as the light source, operating at a wavelength of 1625 nm, where glucose exhibits an overtone absorption band with low water interference. In particular, the glucose overtone absorption band near 1638 nm is primarily attributed to the first overtone of C–H stretching vibrations, with additional contributions from O–H and C–O combination bands [7,21]. The maximum output power in pulsed mode was approximately 80 mW, with a pulse width ranging from 10 to 33 μs. The laser was operated at 25 °C with a threshold current of 60 mA. The driving current was frequency-modulated between 7 and 40 kHz using square waves with a 40% duty cycle, generated by a function generator (Agilent 55321A, Santa Clara, CA, USA). The emitted laser beam was collimated and focused using optical lenses to achieve a spot size of less than 2 mm at the focal point. The laser beam was then directed to the center of the PA cell cavity, where samples were placed. The convex lens was used to focus the laser beam to the focal point in the PA cell to ensure that the laser beam passes through the cavity without light reflections on the sides of the cavity. It is necessary to avoid light reflections inside the cavity to reduce reflection-induced background signals. In order to regulate chamber humidity and prevent condensation on the skin phantoms during measurements, a ventilation system with N_2_ flow was used.

**Figure 3:**
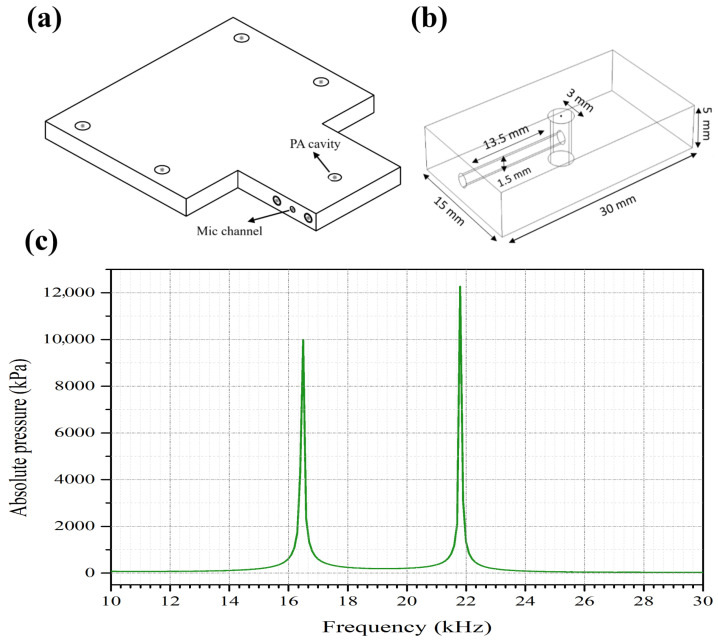
(**a**) PA cell sketch. (**b**) PA cavity dimensions. (**c**) Simulated resonance frequencies of the PA cell.

The acoustic cell, as shown in Figure 3, was designed and simulated in COMSOL Multiphysics, version 6.1 [43] for amplification and collection of acoustic signals. The PA cell featured a cavity with a diameter of 3 mm and a length of 5 mm, while the microphone channel measured 13.5 mm in length and 1.5 mm in diameter. Thereafter, the cell was fabricated using oxygen-free copper and the cell surface was gold-electroplated to prevent oxidation, which helps maintain optimal thermal conductivity. The theoretical resonance frequencies of the cell were computed in simulation to be 16.5 and 21.8 kHz, both with high Q factors. A slight change in resonance frequency is expected during the in vivo and in vitro experiments due to the applied pressure on human fingers or solid samples. An analog acoustic sensor (SPU0410LR5H-QB, DigiKey, Thief River Falls, MN, USA) was mounted on the side wall of the acoustic cell to record amplified acoustic signals. The acoustic sensor exhibits a strong acoustic response in the frequency range of interest, aligning with the resonance frequencies of the acoustic cell.

To improve the signal-to-noise ratio (SNR), the integrated NIR–PA system was calibrated and optimized prior to glucose measurements. First, the laser was switched off to measure the baseline noise signals across 7–40 kHz. Next, the laser was turned on and operated without the convex lens to evaluate background signals. The lens was then installed to focus the beam at the focal point of the PA cavity, reducing light reflections on the cavity walls. For system calibration, a carbon plate was placed on the cavity to verify the detected acoustic signals. The system was then tested by measuring a human index finger and glucose phantoms to analyze the characteristics of the generated signals.

**Figure 4:**
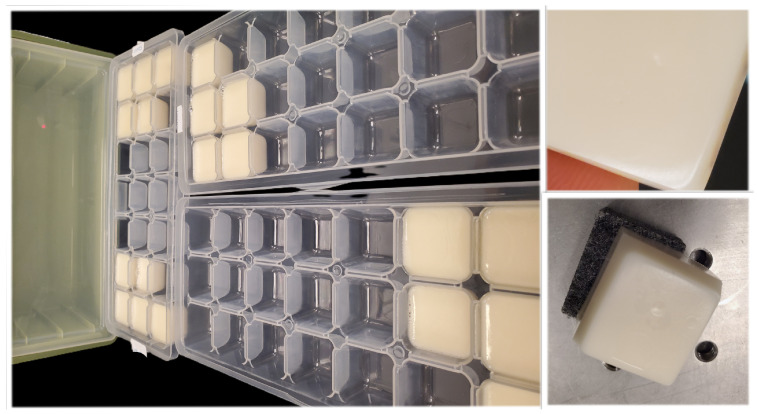
Glucose skin phantoms.

Two sets of skin phantoms with varying glucose concentrations were prepared as test models for the system. Each set included 12 phantoms with glucose concentrations ranging from 85 to 250 mg/dL in steps of ±15 mg/dL, covering both normal and high blood glucose ranges. The phantoms were prepared following Lazebnik et al. [44] and Aloraynan et al. [2], with dimensions of approximately 2×2×2 cm. Their optical properties in the infrared region were verified to be comparable to human skin [2]. The glucose gradient of ±15 mg/dL was designed to align with FDA detection requirements. All ingredients, including glucose, were purchased from Sigma-Aldrich (Oakville, ON, Canada). Figure 4 shows samples of glucose skin phantoms before and after measurements. Before measurements, the phantoms were stored in sealed containers to prevent dehydration. More details on phantom preparations are provided in [2].

The glucose detection measurements were conducted for both sets of samples on two consecutive days. Each glucose sample in the first set was individually placed over the PA cell cavity at room temperature. A micro linear actuator (FA-MU-8-12-1, Firgelli Automations, Surrey, BC, Canada) was installed to apply a well-controlled pressure of around 6 N/cm^2^ on the top of the phantoms. The applied pressure was further monitored with a pressure sensor (400 FSR, Interlink Electronics, Fremont, CA, USA) that was placed on the PA cell beneath the samples, without interfering with the PA cavity. The pressure sensor has an active circumference of 5.1 mm and was connected to a multimeter to measure the applied pressure level. Maintaining a constant pressure during measurements plays a critical role in ensuring high reproducibility of the measurements [45]. Moreover, the temperature during the measurement was maintained constant to avoid fluctuations in the collected signals. Table 2 shows the summary of the two-day measurement procedure.

The fiber-coupled, modulated beams of the 1625 nm NIR laser were focused at the center of the PA cavity to generate acoustic waves in the samples. Each of the 12 glucose phantoms, ranging from 85 to 250 mg/dL, was scanned over the frequency range of 12–40 kHz with a frequency step of 0.15 kHz. The NIR light exhibits a high penetration depth into the samples, allowing acoustic signals to be generated from the inner glucose molecules. The PA cavity amplified the generated acoustic waves, which were then collected by the PA acoustic sensor and transmitted to a low-noise lock-in amplifier (SR830, Stanford Research Systems, Sunnyvale, CA, USA) for processing with a time constant of 300 ms. Each phantom was scanned three times across the designated frequency range, with a total measurement time of approximately three minutes per sample. The processed signals were transmitted to a PC via a data acquisition system for further analysis. The glucose detection experiment was repeated the following day with the second set of phantoms under similar procedures. All measurements were conducted in a light-isolated and vibration-reduced environment to minimize external noise interference.

### MachineLearning for Glucose Detection

Despite recent significant advances, machine learning (ML) has not yet been widely applied in NIR and PA spectroscopy for noninvasive glucose detection. ML-based models can enhance detection sensitivity and selectivity in noninvasive glucose applications. Moreover, the application of ML can help address the complexity of glucose detection in the presence of various blood constituents or under inconsistent environmental parameters. In noninvasive optical spectroscopy, ML models can also be designed to discriminate glucose-related signals despite variations in human skin conditions for in vivo measurements. As a result, ML models can lead to the development of universal detection models for noninvasive applications.

Both classification and regression techniques can be applied in noninvasive glucose detection [46,47,48]. Classification approaches produce discrete outputs associated with predefined classes, whereas regression models provide quantitative predictions. Specifically, classification models produce discrete glucose categories which help to cover wider glucose ranges, while regression methods estimate continuous glucose concentration values [49,50,51]. Thus, reducing the glucose concentration differences between discrete categories improves prediction sensitivity in the prepared phantoms [52].

Different ML-based classification models were investigated in order to detect glucose levels in the phantom using the collected acoustic signals. According to the detection accuracy of the investigated models, the KNN and WNN were chosen for glucose detection. The new class prediction of the KNN algorithm relies on the proximity of this class to another predefined one within the feature space, in a specified number K of nearest neighbours. In the WNN, the network has many neurons in its hidden layers, which are fewer compared to deep networks. The WNN models focus on learning specific patterns and interactions in their predictions.

Different ML-based classification models were investigated in order to detect glucose levels in the phantoms using the collected acoustic signals. Based on comparative preliminary tests, the KNN and WNN models were selected because they provided the best balance between prediction accuracy and model simplicity for the available dataset size. All 187 features in the frequency-domain extracted from each measurement round were used as input to the models, and no manual elimination of features or reduction in dimension was applied. This choice was made to evaluate the information content of the raw acoustic spectra directly.

In order to generate sufficient data for ML training, each glucose sample was scanned three times over a frequency range of 12–40 kHz with a frequency step of 150 Hz. These measurements were repeated over another day using different samples, resulting in six observations per glucose concentration, each represented by a 187-feature vector. In total, 72 observations were obtained across the 12 glucose levels, corresponding to 13,464 scalar data points. Generating a large dataset supports effective ML model training, while appropriate data organization plays a critical role in model performance. In ML, each column corresponds to a feature, and each row represents an individual observation. Thus, the values within each column have to be correlated to define a significant feature for the algorithm. In this study, data points corresponding to each frequency were assigned to a single column, forming a distinct feature associated with a given class label. In other words, each measurement round (12:0.15:40 kHz) was converted into a vector before being combined with other rounds into a single matrix. This data structure resulted in 187 features with 6 rounds per glucose class, as summarized in Table 3.

The model was evaluated using 10-fold cross-validation. The dataset was divided into ten approximately equal subsets. In each iteration, one fold was used as the validation set to assess the model performance, while the remaining nine folds were used for training. This procedure was repeated until each fold had served as the validation set once. In addition, 10% of the data was held out for post-training evaluation of the trained model.

## 3. Results and Discussion

**Figure 5:**
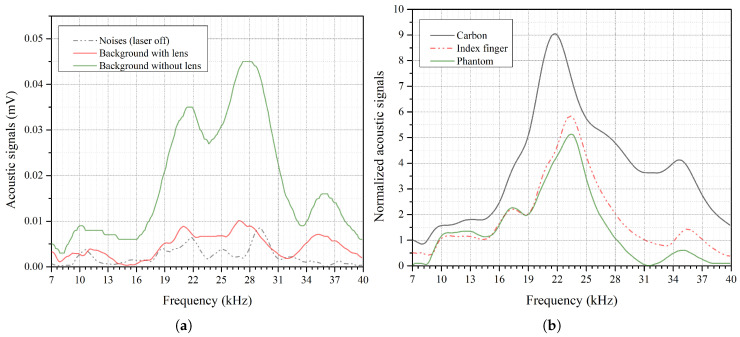
(**a**) Background spectra with and without the focusing lens (laser on) and noise spectra (laser off). (**b**) Acoustic spectrum for carbon, index finger and phantom.

Figure 5a shows the collected acoustic spectrum for noise and background signals. The noise spectrum was recorded by the microphone for the modulation range while the laser was off, yielding an average of 2.3 μV over the entire spectrum range. Before installing the lens, the laser was turned on, and the average background signal rose to 18.6 μV, with a maximum of 45 μV. These elevated background signals are due to the high divergence of the NIR light beam inside the resonator cavity of the PA cell, which has a diameter of 3 mm. Therefore, it was necessary to install a convex lens to focus the laser beam to a diameter of less than 3 mm at the focal point to avoid light reflection inside the PA cavity. Hence, installing the NIR lens reduced the average background signal to 4.5 μV, with a maximum of 10.1 μV. Reducing background signals enables accurate glucose detection measurements. Eventually, the system achieved an SNR of 8.15 for the range of 7–40 kHz and 8.71 for the range of 12–40 kHz for phantom measurements. The maximum SNR level exceeded 30 at the second resonance frequency of the PA cell. Thus, the second resonance frequency can be more sensitive to glucose differences.

The system was then calibrated using a thin carbon plate to enhance the acoustic signal generated and collected by the NIR and PA setup. Carbon exhibits uniform and high optical absorption in the NIR region to ensure the system provides strong and consistent PA signals. Figure 5b shows the normalized acoustic spectrum of the carbon plate compared to that of a human index finger and a phantom sample. The carbon plate recorded a high acoustic signal, with an average of 306 μV and a maximum of 724 μV at the resonance frequency of 21.8 kHz. The average SNR exceeded 200 in the carbon calibration. Subsequently, the PA spectra of a human index finger and a phantom sample were acquired to evaluate their spectral similarity. As illustrated in Figure 5b, the two spectra exhibited comparable characteristics, with mean amplitudes of 18.8 μV and 14.5 μV, and peak values of 58.5 μV and 51.3 μV for the index finger and phantom, respectively. Both values were obtained at the same resonance frequency of 23.4 kHz. The shift in the resonance frequency of the index finger and the phantom compared to carbon is due to the difference in material properties. Furthermore, the arch of the bottom surface of both the index finger and the phantom may cause a slight shift in the resonance frequency. The phase shifts among the sample spectra must be rectified to a specific frequency for a precise comparison of glucose concentration differences.

**Figure 6:**
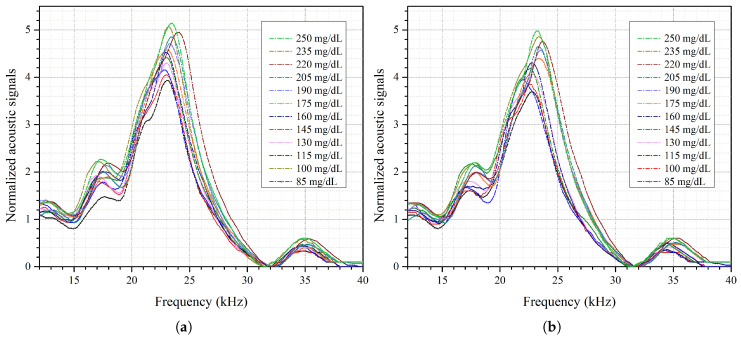
(**a**) Acoustic spectrum of each glucose skin sample from 85 to 250 mg/dL for day 1. (**b**) Acoustic spectrum of each glucose skin sample from 85 to 250 mg/dL for day 2.

**Figure 7:**
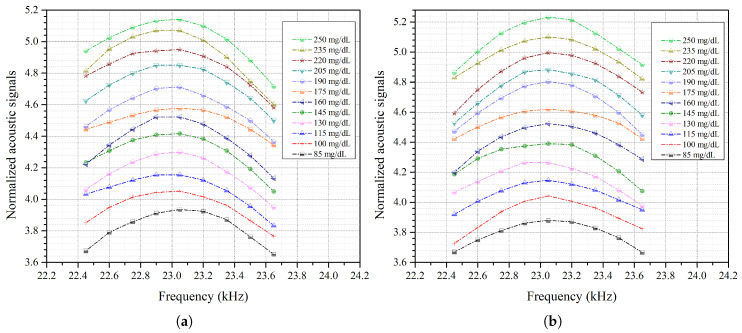
(**a**) Rectified acoustic spectrum for day 1. (**b**) Rectified acoustic spectrum for day 2.

Figure 6a,b show the acoustic spectrum of glucose phantoms, ranging from 85 to 250 mg/dL, for the first and second measurements, respectively. The figures show the average of three rounds of measurements for each glucose phantom using a single NIR laser. The acoustic spectrum of the second peak, ranging from 22 to 24 kHz, was found to be consistent and sensitive to glucose concentrations in the phantoms. The peak value of each glucose phantom spectrum was then centered at 23.05 kHz, for both days, as shown in Figure 7a,b. After the peak was centered, only acoustic signals of the nine-value peak-centered spectral window were integrated to determine the difference in glucose levels. In spectral analysis, the half-power method is commonly applied to determine the bandwidth of a resonance. Here, features were extracted from the dominant spectral peak and its immediate neighbor points only due to the relatively low acoustic signal generated by the NIR laser. Specifically, a peak-centered window consisting of a peak value and four adjacent points on each side was used for analysis. These features were extracted without further data processing or using multivariate algorithms. The dominant spectral peak method is useful when the differences in signals are small or the SNR is low. Hence, this approach is effective for applications with low-power NIR sources to detect small variations in glucose concentrations.

**Figure 8:**
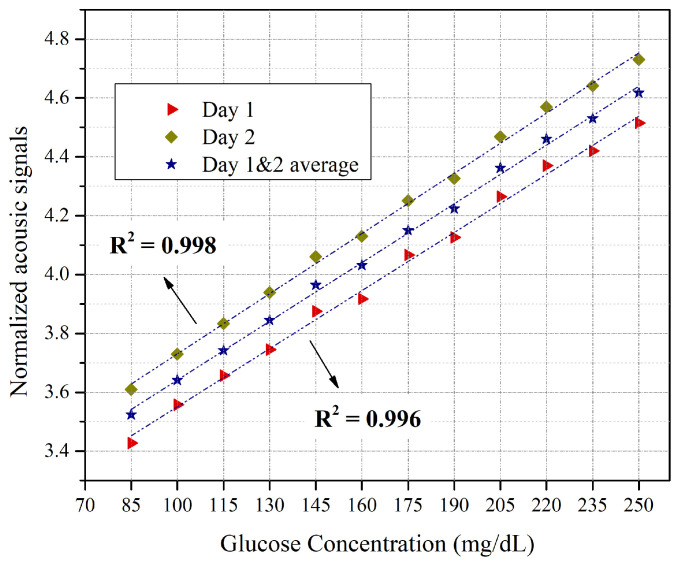
Relationships between the acoustic signals and the corresponding glucose samples for the two-day measurements.

After extracting the nine-value peak-centered spectral window, the area under each curve was integrated to demonstrate the relationship between the acoustic signal and the corresponding glucose phantoms for both days. Figure 8 shows the correlation of glucose concentrations to acoustic signals for day one, day two, and the average for both days. It shows that the NIR and PA system was able to distinguish the differences in glucose concentration for both measurement days. The linear correlation factors for day one and day two were 0.996 and 0.998, respectively. This gives an average linear correlation of 0.997 for both days. The average difference in acoustic signals between glucose phantoms for the first day was 2.54% for all concentrations, with an average standard deviation of 1.01. The average difference improved to 2.69% on the second day, with an average standard deviation of 0.51. Table 4 shows the average differences in acoustic signals between glucose phantoms for the two-day measurements. These differences indicate the system’s sensitivity in distinguishing glucose concentrations in the skin-like phantoms. However, the fluctuations in the differences can be attributed to possible variations in the glucose phantoms and to the attenuation of the collected signals due to the propagation distance. Nevertheless, the results demonstrate a low standard deviation for the entire set, indicating high reliability of the measurements. These findings show that the NIR and PA system was able to detect differences in glucose levels for skin-like phantoms using a single NIR wavelength of 1625 nm. Furthermore, the detection sensitivity of the NIR and PA system was enhanced to ±15 mg/dL to fulfill the FDA [41] and Health Canada requirements [42]. Nevertheless, it should be noted that the current study is limited to homogeneous skin-mimicking phantoms and does not account for physiological variability present in real tissue.

### Glucose Detection Using Machine Learning

In the previous results, ML models were not involved to achieve the correlation between the acoustic signals and glucose concentrations to determine the system’s feasibility. ML models can assist in enhancing prediction sensitivity by extracting subtle glucose-related patterns from PA signals that are often obscured by noise or weak absorption features. Also, the selectivity of the system in the presence of other blood components can be improved using ML models. In this work, KNN and WNN classification models were developed and trained using the collected acoustic signals of the glucose phantoms. The data used during training was not preprocessed or rectified. Although glucose concentration is inherently a continuous variable, a classification framework was adopted in this study to evaluate the discriminative detectability of the system. The class interval of ±15 mg/dL reflects the spacing between glucose levels rather than the true analytical resolution of the system.

**Figure 9:**
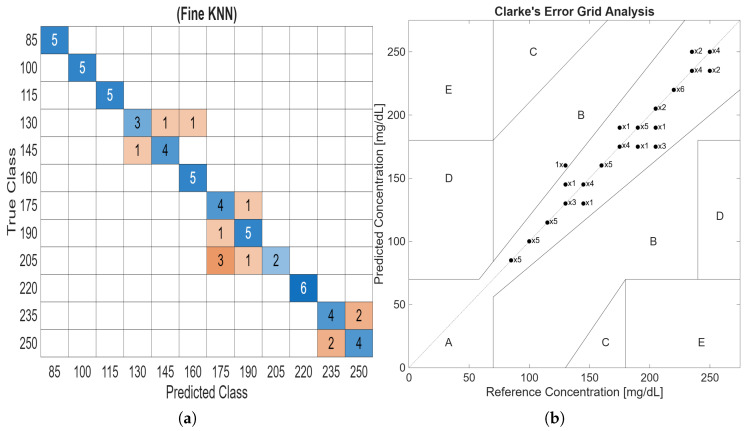
(**a**) Confusion matrix of the KNN model. (**b**) Clarke’s EGA of the KNN prediction model.

The KNN classifier achieved an average prediction accuracy of 80.0% for all classes and an F1 score of 79.9%. The Fine KNN used one neighbour for prediction with Euclidean distance. All of the 187 features were used to train the KNN model to detect glucose in the samples using the collected acoustic signals. Subsequently, the trained model correctly predicted the class of the test data with an accuracy of 91.7%. The prediction accuracy of the model can be improved further by increasing the size of the dataset and applying multivariate algorithms. The produced confusion matrix of the KNN classifier is shown in Figure 9a. The confusion matrix illustrates the performance of the classifier by corresponding true classes with the predicted classes. The main diagonal of the confusion matrix represents the number of samples that are correctly classified.

The U.S. Food and Drug Administration requires that at least 99% of predicted glucose measurements lie within zones A and B of Clarke’s EGA [41], which is a standard metric for evaluating the clinical accuracy of glucose predictions against reference measurements. In order to assess the model’s prediction accuracy, the confusion matrix was transformed into Clarke’s EGA, as illustrated in Figure 9b. This representation indicates the frequency with which the classifier predicts each glucose class to the reference concentration. The results show that 98.4% of the predicted results fall within zone A, while only 1.6% are located in zone B. The classifier has no predicted results in zones C, D, and E.

**Figure 10:**
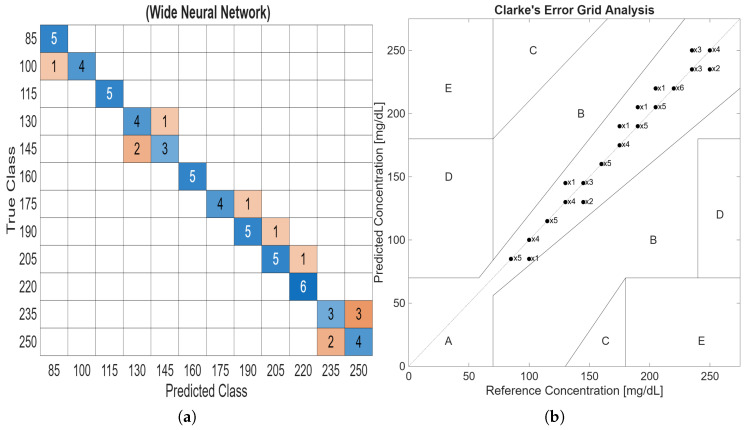
(**a**) Confusion matrix of the WNN model. (**b**) Clarke’s EGA of the WNN prediction model.

The WNN classifier model achieved a prediction accuracy of 81.5% and an F1 score of 81.9%. Similar to the KNN model, all 187 features were used as inputs. The WNN architecture consisted of one fully connected hidden layer with 100 neurons. The WNN-trained model precisely predicted all the test data that was reserved for post-training evaluation. The produced confusion matrix of the WNN classifier is shown in Figure 10a. All of the WNN predicted results fall in zone A of Clarke’s EGA as shown in Figure 10b. Although WNN has an improved detection precision, both models satisfy the FDA requirements.

## 4. Conclusions

An NIR–PA system was developed for convenient noninvasive blood glucose detection. A single-wavelength SWIR laser operating at the glucose overtone wavelength of 1625 nm was employed as the photoacoustic excitation source. Artificial biomedical skin phantoms with properties similar to those of human skin were prepared as test models for the system. The phantoms were prepared to represent normoglycemic and hyperglycemic blood glucose levels with glucose concentration intervals of ±15 mg/dL. The dominant spectral peak-based feature extraction approach significantly enhances the recognition of glucose-related characteristics in the PA spectrum. As a result, a linear correlation of 0.997 was achieved for two-day measurements. The detection sensitivity of the NIR–PA system was improved to ±15 mg/dL using a single-wavelength laser. These results were obtained without the need for advanced algorithms or extensive post-processing of the data. These results demonstrate that clinically relevant glucose sensitivity can be achieved under controlled phantom conditions using a single-wavelength NIR–PA configuration without relying on extensive spectral preprocessing. Although this study focused on glucose-dominant samples, future investigations should evaluate the influence of other endogenous components near 1625 nm such as albumin and lipids to further assess the selectivity of the proposed NIR–PA approach.

KNN and WNN ML models have been developed to classify glucose levels in the phantoms with 13,464 data points. The models were trained on unprocessed data with 187 features. Prediction accuracies of 80.0% and 81.5% were achieved by the KNN and WNN classifiers, respectively. Both models placed 100% of the predictions results within Zones A and B of Clarke’s EGA. These findings satisfy the FDA and Health Canada standards for glucose monitors. The prediction accuracy can be further enhanced by increasing the dataset size in future measurements and using advanced algorithms such as Bayesian Regularized Artificial Neural Networks (BRANN) and ensemble learning.

In practical applications, NIR–PA measurements may be influenced by various physiological and environmental factors, including skin thickness, temperature fluctuations, and hemodynamic variations. These factors can alter both optical absorption characteristics and acoustic signal generation. An investigation of these effects will be considered in future works for planned in vivo measurements.

## Figures and Tables

**Table 1:** Absorption peaks of glucose in the NIR regime [7].

Wavelength (nm)	Stretching Group
910	4*v*CH
930	3*v*CH_2_
939	3*v*OH
1018	*v*CH combination
1042	*v*CH combination
1126	3*v*CH
1408	2*v*OH
1536	*v*OH + *v*CH
1638	First overtone
1688	2*v*CH
2261	*v*CH + *v*CCH
2273	O-H/C-O combination

*v* denotes stretching.

**Table 2:** Summary of measurement procedures for glucose detection.

Index	Sample No.	Glucose Level	Round 1	Round 2	Round 3
Day 1	1st sample	85 mg/dL	12:0.15:40 kHz	12:0.15:40 kHz	12:0.15:40 kHz
Day 2	2nd sample	85 mg/dL	12:0.15:40 kHz	12:0.15:40 kHz	12:0.15:40 kHz
Day 1	1st sample	100 mg/dL	12:0.15:40 kHz	12:0.15:40 kHz	12:0.15:40 kHz
⋮	⋮	⋮	⋮	⋮	⋮
Day 2	2nd sample	235 mg/dL	12:0.15:40 kHz	12:0.15:40 kHz	12:0.15:40 kHz
Day 1	1st sample	250 mg/dL	12:0.15:40 kHz	12:0.15:40 kHz	12:0.15:40 kHz
Day 2	2nd sample	250 mg/dL	12:0.15:40 kHz	12:0.15:40 kHz	12:0.15:40 kHz

**Table 3:** Data organization of the glucose acoustic spectra for ML training.

Index	12 kHz	12.15 kHz	12.30 kHz	…	23.35 kHz	23.50 kHz	…	40 kHz	Class Label
Day 1	round 1	round 1	round 1	…	round 1	round 1	…	round 1	85 mg/dL
	round 2	round 2	round 2	…	round 2	round 2	…	round 2	85 mg/dL
	round 3	round 3	round 3	…	round 3	round 3	…	round 3	85 mg/dL
Day 1	round 1	round 1	round 1	…	round 1	round 1	…	round 1	100 mg/dL
	round 2	round 2	round 2	…	round 2	round 2	…	round 2	100 mg/dL
	round 3	round 3	round 3	…	round 3	round 3	…	round 3	100 mg/dL
.	.	.	.	…	.	.	…	.	.
.	.	.	.	…	.	.	…	.	.
.	.	.	.	…	.	.	…	.	.
Day 1	round 1	round 1	round 1	…	round 1	round 1	…	round 1	250 mg/dL
	round 2	round 2	round 2	…	round 2	round 2	…	round 2	250 mg/dL
	round 3	round 3	round 3	…	round 3	round 3	…	round 3	250 mg/dL
Day 2	round 1	round 1	round 1	…	round 1	round 1	…	round 1	85 mg/dL
	round 2	round 2	round 2	…	round 2	round 2	…	round 2	85 mg/dL
	round 3	round 3	round 3	…	round 3	round 3	…	round 3	85 mg/dL
.	.	.	.	…	.	.	…	.	.
.	.	.	.	…	.	.	…	.	.
.	.	.	.	…	.	.	…	.	.
Day 2	round 1	round 1	round 1	…	round 1	round 1	…	round 1	250 mg/dL
	round 2	round 2	round 2	…	round 2	round 2	…	round 2	250 mg/dL
	round 3	round 3	round 3	…	round 3	round 3	…	round 3	250 mg/dL

**Table 4:** Differences in acoustic signals between glucose levels.

**Glucose samples (mg/dL)**	250–235	235–220	220–205	205–190	190–175	175–160	160–145	145–130	130–115	115–100	100–85
**Differences (%)**	2.08	1.89	2.37	2.63	1.99	3.44	1.92	3.30	2.52	3.12	3.56

## Data Availability

The raw data supporting the conclusions of this article will be made available by the authors on request.
